# Olfactory Bulb Muscarinic Acetylcholine Type 1 Receptors Are Required for Acquisition of Olfactory Fear Learning

**DOI:** 10.3389/fnbeh.2019.00164

**Published:** 2019-07-19

**Authors:** Jordan M. Ross, Mounir Bendahmane, Max L. Fletcher

**Affiliations:** ^1^Department of Anatomy and Neurobiology, University of Tennessee Health Science Center (UTHSC), Memphis, TN, United States; ^2^Department of Pharmacology, University of Michigan, Ann Arbor, MI, United States

**Keywords:** olfaction, fear learning, acetylcholine, muscarinic, olfactory bulb, pharmacology, behavior

## Abstract

The olfactory bulb (OB) receives significant cholinergic innervation and widely expresses cholinergic receptors. While acetylcholine (ACh) is essential for olfactory learning, the exact mechanisms by which ACh modulates olfactory learning and whether it is specifically required in the OB remains unknown. Using behavioral pharmacology and optogenetics, we investigated the role of OB ACh in a simple olfactory fear learning paradigm. We find that antagonizing muscarinic ACh receptors (mAChRs) in the OB during fear conditioning but not testing significantly reduces freezing to the conditioned odor, without altering olfactory abilities. Additionally, we demonstrate that m1 mAChRs, rather than m2, are required for acquisition of olfactory fear. Finally, using mice expressing channelrhodopsin in cholinergic neurons, we show that stimulating ACh release specifically in the OB during odor-shock pairing can strengthen olfactory fear learning. Together these results define a role for ACh in olfactory associative learning and OB glomerular plasticity.

## Introduction

The olfactory bulb (OB) receives significant input from neuromodulatory centers that release norepinephrine, serotonin, and acetylcholine (ACh) into the OB, which can alter olfactory learning and associated plasticity (Wilson et al., 2004; Fletcher and Chen, 2010; Ross and Fletcher, 2019). In particular, the OB is densely innervated by cholinergic projection neurons from the basal forebrain (Macrides et al., 1981; Záborszky et al., 1986). The cholinergic projection neurons terminate densely in the glomerular layer (Shipley and Ennis, 1996) where odor information is first processed in the brain and represented in a spatiotemporal pattern of glomerular activation unique to each odor (Wachowiak and Cohen, 2001; Spors and Grinvald, 2002; Bozza et al., 2004; Mori et al., 2006; Fletcher et al., 2009; Storace and Cohen, 2017). Both nicotinic and muscarinic ACh receptor (mAChR) subtypes are also widely expressed in the glomerular layer (Le Jeune et al., 1995; Castillo et al., 1999; Ghatpande and Gelperin, 2009; D’Souza and Vijayaraghavan, 2012) and have varied consequences on OB processing (Ravel et al., 1990; Elaagouby et al., 1991; Castillo et al., 1999; Liu et al., 2015; Smith et al., 2015; Case et al., 2017), providing distinct mechanisms by which ACh can modulate olfactory information.

ACh is crucial to olfactory function (Fletcher and Wilson, 2003; Bendahmane et al., 2016; Linster and Cleland, 2016; Chan et al., 2017; Ogg et al., 2018), and disruption of ACh signaling is known to affect olfactory appetitive learning across species (Ravel et al., 1994; Mandairon et al., 2006; Chaudhury et al., 2009; Devore et al., 2012; Hellier et al., 2012; Williamson and Wright, 2013; Chan et al., 2017); however, little is known regarding the extent to which cholinergic signaling affects aversive olfactory learning. Recent reports demonstrate that olfactory fear conditioning induces OB plasticity (Fletcher, 2012; Kass et al., 2013; Kass and McGann, 2017; Ross and Fletcher, 2018b), yet we lack a mechanistic understanding of the causes of such alterations. Modeling data demonstrates ACh release during olfactory learning increases mitral cell (MC) synchrony and facilitates synaptic plasticity in piriform cortex (PCx), leading to enhanced learning (de Almeida et al., 2013). In line with this, systemically inhibiting cholinergic signaling during acquisition disrupts olfactory fear learning (Kroon and Carobrez, 2009; Silva et al., 2015) but does not impair olfactory perception (Doty et al., 2003; Pavesi et al., 2012) nor alter sensitivity to unconditioned stimuli (Anagnostaras et al., 1999). Together, this demonstrates ACh is required for olfactory fear learning, possibly by enabling plasticity required for learning associations between the conditioned stimulus (CS) and unconditioned stimuli during acquisition of fear learning. Considering ACh is required for olfactory fear learning and its widespread innervation of the olfactory system, its role in olfactory fear learning and plasticity presents an interesting target for further investigation.

Although it has been determined that olfactory fear learning is mediated by muscarinic, not nicotinic, AChRs (Pavesi et al., 2012), the systemic nature of mAChR antagonism makes it difficult to conclude whether the decreased fear learning was due antagonism of mAChRs in olfactory regions or other affected brain regions, such amygdala or PCx which also express mAChRs (Spencer et al., 1986; Buckley et al., 1988). Modeling suggests mAChRs regulate synaptic plasticity in PCx but also increase MC synchrony in the OB, which could lead to enhanced PCx learning (Hasselmo and Barkai, 1995; de Almeida et al., 2013), making it important to establish whether mAChRs are required specifically in the OB during olfactory fear conditioning for learning to occur. Furthermore, there are two subtypes of mAChRs expressed widely in the OB and use of broad mAChR antagonists makes it unclear which subtypes are necessary for olfactory fear learning. While cholinergic signaling through mAChRs appears necessary for fear learning, the role it plays within the OB during associative learning has yet to be determined.

Here, we use a combination of behavioral pharmacology and optogenetics to characterize the role of OB ACh in olfactory fear learning. To determine the extent to which OB muscarinic cholinergic signaling supports fear conditioning we directly infused scopolamine (SCOP), a mAChR antagonist, into the OB during fear conditioning. When tested 24 h later, mice in which mAChRs were inhibited during odor-shock pairing, exhibit significantly reduced learned fear to the CS. By infusing specific antagonists of different mAChRs directly into the OB during olfactory fear conditioning, we identify that activation of the m1 subtype, but not the m2 subtype, of mAChRs in the OB is necessary for acquisition of olfactory fear learning. Furthermore, we use mice expressing channelrhodopsin in cholinergic neurons to stimulate the release of ACh specifically in the OB during olfactory fear conditioning and demonstrate that enhanced OB ACh can strengthen olfactory fear learning. This establishes that OB ACh can bidirectionally modulate the strength learning. Finally, we subject mice to an odor investigation task under the influence of OB SCOP and find that inhibition of mAChRs does not alter olfactory perception, and therefore cannot be the cause of suppressed learning. Altogether these results define a role for ACh in olfactory associative learning and OB glomerular plasticity.

## Materials and Methods

### General Methodology

#### Animals

A total of 93 mice were used. OB cannula experiments were performed using adult male and female C57BL6/J (Jax Stock no: 000664) mice (*n* = 78). Optogenetic experiments were performed on adult male and female B6.Cg-Tg(Chat-COP4*H134R/EYFP, Slc18a3)6Gfng/J (ChAT-ChR2+) and wild-type (ChAT-ChR2−) littermates (Jax Stock No: 014546) mice (*n* = 15). All experimental protocols were approved by the University of Tennessee Institutional Animal Care and Use Committee.

#### Surgical Procedures

For all surgical procedures, mice were anesthetized under ketamine/xylazine (100/10 mg/kg, i.p.) and given carprofen (5 mg/kg, s.c.) after depth of anesthesia was verified by tail pinch. Mice were secured in a stereotaxic device and maintained on a heating pad for the duration of the surgery. All mice were implanted with a stainless steel anchor screw in the parietal bone to help secure cannula/LED to the skull. Mice used for cannula experiments (Experiments 1 and 3) received stainless steel bilateral cannula (Plastics One; C235GS-5-2.0/SPC) implanted in the OBs (Bregma: 4.2 mm anterior, 1 mm lateral on either side, 1 mm ventral). At the end of the surgery, a dummy and cap (Plastics One; C235DCS-5/SPC and 303DC/1B) were inserted into the cannula of cannulated mice. Mice used for optogenetic experiments were implanted with miniature blue LEDs (Osram; LBW5SN), following thinning of the bone overlying the OBs with a dental drill (Ogg et al., 2018). Mice were given at least 1 week to recover prior to experimentation.

#### Drugs

For cannulated mice, 0.5 μl drug or vehicle (VEH) infusions were delivered bilaterally at a rate of 0.125 μl/min. Infusion cannula were left in place for 2 min following delivery to allow for diffusion. Mice received one of the following infusions either before training or before testing: non-selective muscarinic receptor antagonist SCOP hydrobromide (SCOP; Sigma-Aldrich, cat. no: S0929), selective muscarinic m1 receptor antagonist pirenzepine dihydrochloride (PIR; Tocris Bioscience, cat. no: 1071), selective muscarinic m2 receptor antagonist AF-DX 116 (AFDX; Tocris Bioscience, cat. no: 1105), or an appropriate VEH [Ringer’s solution or dimethyl sulfoxide (DMSO; Sigma-Aldrich, cat. no: D8418)].

#### Olfactory Fear Conditioning and Testing

Olfactory fear conditioning was carried out as previously described (Ross and Fletcher, 2018a). Briefly, animals were trained in a single-day classical fear conditioning paradigm where six 10 s presentations of a single odor, ethylvalerate (E5; Sigma-Aldrich, cat. No: 290866) diluted to ~200 ppm in mineral oil co-terminated with a 0.6 mA, 0.5 s foot shock. Mice were allowed to acclimate to the training chamber for 10 min before training began. Twenty-four hours following training, mice were placed in a separate testing context and given 10 min to acclimate before they were assessed for behavioral fear to the CS. Fear was measured by behavioral freezing, a widely used measure of fear (Blanchard and Blanchard, 1969a,b; Fanselow, 1980), which is characterized by cessation of voluntary movement. Testing consisted of two 20 s presentation of E5 (ITI = 3 min), starting in the second minute of the test session. Freezing bouts, lasting a minimum of 2 s, were calculated using FreezeFrame4 (Coulbourn Instruments), and binned into 60 s segments, to be reported as % of time spent freezing during the 60 s bin in which odor was present.

#### Odor Investigation

Mice with bilateral OB cannula were placed in a standard shoebox cage (18.4 cm *W* × 29.2 cm *D* × 12.7 cm *H*) devoid of bedding placed inside an open field chamber (40 cm *W* × 40 cm *D* × 35 cm *H*; Stoelting). Air or air odorized by 1% s.v. isoamylacetate (Sigma-Aldrich, cat. no: W205508) was constantly delivered to the chamber through tubing along one of the corners. The advantage of this paradigm is that it allowed for odor delivery without experimenter inference, mouse disruption, or visual/auditory cues that could result in unintended behavioral effects. A vacuum pulled air away through small holes in the chamber to prevent odor build-up. A video camera was positioned towards the side of the behavioral chambers and investigative behavior, defined as active sniffing with a raised head, was manually scored using ANY-maze (Stoelting). Ten minutes prior to placement in the chamber, mice received infusions of either 1 mM SCOP or VEH. Mice were given 10 min to (with non-odorized air) before presentation of odorized air. Investigation behavior was scored for the final 120 s of the acclimation period and the first 60 s of the odor presentation.

#### Optogenetic Stimulation

Prior to placement in the training chamber, head-mounted LEDs were connected to a pulse generator using flexible, light-weight wires. Optogenetic stimulation occurred only during olfactory fear conditioning. The pulse generator delivered a 3 s, 50 Hz train starting 7.5 s after odor onset, such that the stimulation spanned the final 2.5 s of the odor presentation and the 0.5 s foot shock. The stimulation parameters were based on previous laboratory experiments (Ogg et al., 2018). The genetic identity of mice (ChAT-ChR2− vs. ChAT-ChR2+) was not known until after the conclusion of the experiment.

### Detailed Methodology

#### OB Pharmacology

Experiment 1a: cannulated mice received infusions of SCOP (in Ringer’s; 1 μM, *n* = 9; 1 mM, *n* = 7; or 10 mM, *n* = 4) or vehicle (Ringer’s, *n* = 8) prior to training to assess the role of OB muscarinic signaling in acquisition of olfactory fear learning.

Experiment 1b: cannulated mice received infusions of SCOP (in Ringer’s; 1 mM, *n* = 6) or vehicle (Ringer’s, *n* = 8) prior to testing to determine the extent to which OB muscarinic signaling is necessary for expression of learned fear.

Experiment 1c: cannulated mice received infusions of PIR (in Ringer’s; 1 mM, *n* = 7), AFDX (in DMSO; 1 mM, *n* = 7), or vehicle (Ringers, *n* = 7 and DMSO, *n* = 7, respectively) prior to training to ascertain the role of specific OB muscarinic receptors in the acquisition of olfactory fear conditioning.

#### Optogenetic OB Stimulation

Experiment 2: ChAT-ChR2+ (*n* = 9) mice and their wildtype littermates (ChAT-ChR2−; *n* = 5) received optogenetic stimulation of OB cholinergic fibers during olfactory fear conditioning to evaluate the extent to which enhanced OB ACh during odor-shock pairing modulates olfactory fear learning.

#### Odor Investigation

Experiment 3: cannulated mice received infusions of 1 mM SCOP (in Ringers; *n* = 5) or VEH (*n* = 3) before being placed inside a standard shoebox cage located in an open field chamber. Mice were given 600 s to acclimate. The last 120 s of acclimation were recorded and scored for investigative behaviors, defined as actively sampling by sniffing with a raised head. At the end of the acclimation phase, isoamylacetate was added to the constantly circulating air stream to achieve 1% s.v. acetophenone. Investigation behavior was scored for an additional 60 s. Each mouse was tested only once.

### Quantification and Statistical Analyses

Olfactory fear learning was quantified as behavioral freezing during the test session with FreezeFrame4 automated detection software (Coulbourn Instruments) in the 60 s following odor presentation onset (Pavesi et al., 2012; Ross and Fletcher, 2018a). The CS, E5, was presented to each mouse two times during testing, and the freezing values for each epoch were averaged together to obtain a mean freezing score.

All statistical analyses were performed using Prism software (GraphPad, version 5.03) or SPSS (IBM, version 22). All data were subjected to testing for equal variances and normality. A one-tailed independent samples *t*-test was used to compare behavioral freezing between the two cannulated vehicle groups (Ringer’s vs. DMSO), which revealed no significant behavioral difference (*t*_(12)_ = 0.4709, *p* = 0.6462, *M* = 57.960 ± 5.527 and 54.78 ± 3.868, respectively) between the two vehicle controls. Therefore, Ringer’s and DMSO vehicle controls were combined for analysis in Experiment 1b. ANOVAs were used for behavioral data in Experiments 1a, 1c while a one-tailed *t-test* was used for Experiments 1b, 2, and 3. Dunnett’s *post hoc* testing was performed where appropriate. All data are presented as mean ± SEM.

## Results

### Muscarinic Neurotransmission Is Required in the OB During Acquisition for Olfactory Fear Learning

**Figure 1:**
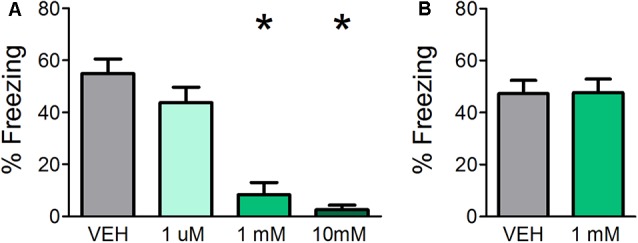
Direct olfactory bulb (OB) application of scopolamine (SCOP) during fear conditioning impairs olfactory aversive fear learning but has no effect on the expression of previously learned fear. **(A)** Mice received infusions of vehicle (VEH) or different concentrations of SCOP (1 μM, 1 mM, or 10 mM), a non-selective antagonist of muscarinic acetylcholine receptors (mAChRs), through cannula directly into the OBs prior to olfactory fear conditioning, in which a single odor (E5) was paired with mild foot shock. Mice were tested for behavioral freezing to the conditioned odor (E5) 24 h later. Mice receiving infusions of 1 mM and 10 mM SCOP demonstrated reduced freezing relative to VEH controls, indicating impaired fear learning when mAChRs are blocked specifically in the OBs. **(B)** Mice were first fear-conditioned to E5. During testing, 24 h after conditioning, mice received direct OB infusions of VEH or 1 mM SCOP. There is no significant difference in behavioral freezing between mice receiving infusions of VEH or 1 mM SCOP, signifying antagonism of mAChRs during expression does not affect olfactory perception or behavioral displays of learned olfactory fear. Data presented as mean ± SEM. *p < 0.05.

Previous experiments demonstrate that muscarinic, but not nicotinic, neurotransmission is required during acquisition of fear learning (Pavesi et al., 2012); however, the use of systemic drug administration could not determine whether it is specifically required in the OB. Therefore, in Experiment 1a, cannulated mice received OB infusions of either VEH or various concentrations of SCOP (1 μM, 1 mM, or 10 mM) in order to test whether blocking muscarinic signaling specifically in the OB during acquisition affects fear learning. All mice were assessed for behavioral freezing to the CS 24 h after training in order to measure fear learning (Figure 1A). VEH infused mice displayed robust behavioral freezing to the CS (mean freezing = 54.92% ± 5.608). Infusions of SCOP prior to training significantly impeded fear learning (*F*_(3,24)_ = 19.724, *p* < 0.0001, *η*^2^ = 0.711). While the lowest SCOP dose (1 μM) did not impact fear learning relative to VEH mice (mean freezing = 43.80% ± 5.9, *p* = 0.309), higher doses of 1 mM and 10 mM significantly decreased freezing (mean freezing = 8.31% ± 4.7, *p* ≤ 0.0001 and mean freezing = 2.55% ± 1.7, *p* < 0.0001, respectively). These results confirm that muscarinic neurotransmission is required for fear learning and establish that it is necessary specifically in the OB during acquisition.

### OB Muscarinic Neurotransmission Is Not Required for Expression of Olfactory Fear Learning

Experiment 1a indicates that OB cholinergic signaling during acquisition is necessary for olfactory fear learning; however, the extent to which muscarinic neurotransmission is required during expression of fear learning is unclear. Therefore, in Experiment 1b, we first subjected mice to olfactory fear conditioning and then infused either SCOP (1 mM, based on the efficacy of 1 mM SCOP in preventing fear learning when administered prior to training in Experiment 1a) of VEH prior to behavioral testing 24 h after training (Figure 1B). Blocking muscarinic receptors *via* OB SCOP infusion (mean freezing = 47.27% ± 5.1) during testing had no effect on behavioral freezing relative to VEH controls (mean freezing = 47.66% ± 5.2; *t*_(12)_ = 0.05283, *p* = 0.4794), indicating OB muscarinic signaling is not required during the expression of a previously learned olfactory fear.

### OB Muscarinic Neurotransmission, Specifically Through mAChR1, Is Required for Fear Learning

**Figure 2:**
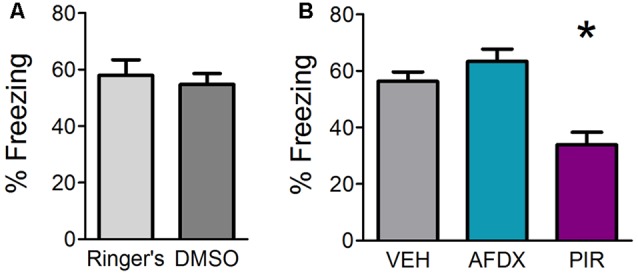
Inhibition of mAChR1, but not mAChR2, decreases behavioral freezing to the conditioned odor. Mice received direct OB infusions of vehicle [VEH, either Ringer’s or dimethyl sulfoxide (DMSO)] or AFDX, a specific antagonist of the m2 subtype of mAChRs, or PIR, a specific antagonist of the m1 subtype of mAChRs, prior to olfactory fear conditioning. The mice were then tested for behavioral freezing 24 h later. **(A)** Mice receiving Ringer’s VEH and those receiving DMSO VEH prior to conditioning do not exhibit different freezing during testing, indicating no difference in learning as a result of the different VEH conditions. **(B)** There is no significant difference in freezing between VEH mice (combined Ringer’s and DMSO) and those receiving infusions of the mAChR2 antagonist AFDX; however, mice treated with PIR before conditioning display reduced freezing relative to VEH mice, suggesting mAChR1 s specifically are required for appropriate acquisition of olfactory fear. Data presented as mean ± SEM. *p < 0.05.

SCOP is a non-selective antagonist of mAChRs. There are two types of mAChRs expressed widely in the OB, mAChR1 and mAChR2. In order to determine which of the receptor subtypes are necessary for fear learning, we infused specific antagonists for either mAChR1 (PIR) or mAChR2 (AFDX) or appropriate VEH in different mice prior to training. Mice receiving Ringer’s VEH and DMSO VEH were combined into a single VEH comparison group after statistical testing revealed no significant difference between the two VEH controls (Figure 2A). Both antagonists for Experiment 1c were delivered at a concentration of 1 mM based on the efficacy of 1 mM SCOP in Experiment 1a. Mice were tested for behavioral freezing, as a measure of learned fear, 24 h after fear conditioning (Figure 2B). Mice receiving VEH infusions prior to training exhibited robust freezing to the CS (mean freezing = 56.37% ± 3.3). As expected, inhibiting mAChRs blocked fear learning (*F*_(2,25)_ = 12.111, *p* = 0.0002, *η*^2^ = 0.4921); however, only infusions of PIR (mean freezing = 33.87% ± 4.5), not AFDX (mean freezing = 63.44% ± 4.3), decreased CS-evoked freezing relative to VEH mice (*p* = 0.001 and *p* = 0.367, respectively). This suggests cholinergic signaling through mAChR1, but not mAChR2, during training is required for olfactory fear learning.

### Stimulating Release of OB ACh During Odor-Shock Pairing Strengthens Olfactory Fear Learning

**Figure 3:**
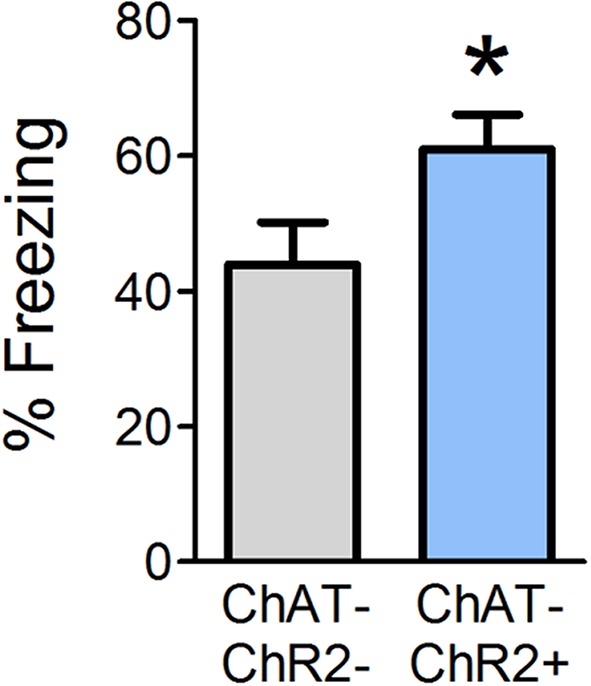
Enhanced OB ACh during odor-shock pairing augments olfactory fear learning. Mice were surgically implanted with a miniature LED directly above the OBs. During olfactory fear conditioning, all mice received light stimulation at the end of each of the six odor-shock pairings. Positive ChAT-ChR2 mice express channelrhodopsin in cholinergic cell populations, such that light stimulation should induce release of ACh in the OBs during odor-shock pairing. When tested 24 h later, ChAT-ChR2+ mice freeze significantly more than ChAT-ChR2− mice, which do not express channelrhodopsin in cholinergic cell populations and should experience no additional ACh release in the OBs as a result of light stimulation. This suggests that increasing OB ACh during olfactory fear conditioning can strengthen fear learning. Data presented as mean ± SEM. *p < 0.05.

Optogenetic OB stimulation has previously been shown to cause behavioral dishabituation of ChAT-ChR2+ but not wild-type (ChAT-ChR2−) littermates (Ogg et al., 2018), consistent with the idea that the stimulation paradigm induces release of ACh into the OB. Given that ACh is necessary during conditioning in order to acquire olfactory fear, we next tested whether supplemental OB ACh could augment fear learning. In Experiment 2, we optogenetically stimulated release of OB ACh specifically during each of the six odor-shock pairings and tested behavioral freezing 24 h later (Figure 3). ChAT-ChR2+ mice displayed augmented freezing (mean freezing = 61.04% ± 5.0) during testing relative to ChAT-ChR2− mice (mean freezing = 43.9% ± 6.3; *t*_(12)_ = 2.077, *p* = 0.030). These results suggest that increasing OB ACh during acquisition of olfactory fear learning can enhance the strength of the learned association.

### Olfactory Investigative Behavior Is Not Affected by Direct OB Antagonism of mAChRs

**Figure 4:**
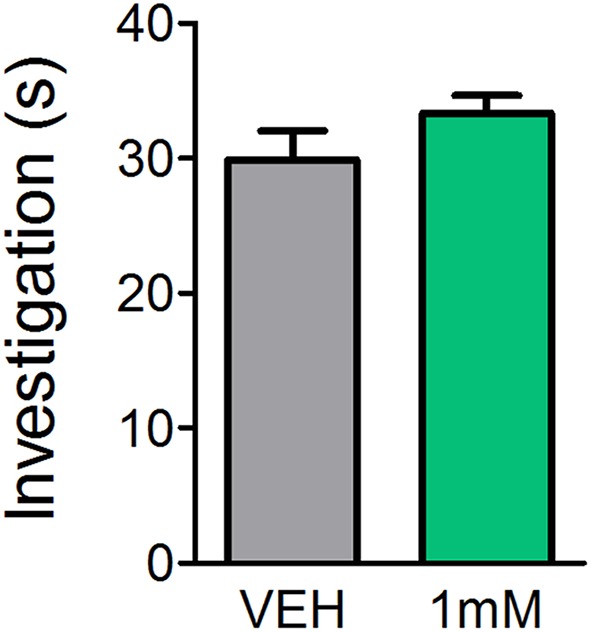
Antagonism of mAChRs does not alter olfactory-driven behaviors. Mice underwent an olfactory investigation paradigm inside an open field chamber to determine whether the mAChR antagonist, SCOP, alters olfactory behaviors. Cannulated mice received direct OB infusions of either 1 mM SCOP or VEH. Time spent performing olfactory investigative behaviors was then scored in response to uncued odor presentations. Mice receiving OB SCOP did not differ from those receiving VEH in terms of time spent investigating. Together, this demonstrates non-specific antagonism of mAChRs does not alter olfactory-driven behaviors or induce temporary anosmia. Data presented as mean ± SEM.

While previous reports indicate mice lacking certain mAChRs exhibit normal basic olfactory investigation (Chan et al., 2017) and systemic administration of the mAChR antagonist SCOP does not impair olfactory perception (Doty et al., 2003; Pavesi et al., 2012), it is unclear whether direct OB application of mAChR antagonists affects olfactory behaviors. In order to determine whether the observed learning impairments were a result of reduced olfactory perception following mAChR antagonism, we subjected mice to an olfactory investigation task in Experiment 3 to assess olfactory function. In Experiment 3 (Figure 4), OB administration of SCOP (1 mM) did not affect investigation of an odorized ball relative to VEH (*t*_(6)_ = 1.483, *p* = 0.0943). Mice spent the same amount of time performing investigatory behaviors during odor presentation regardless of whether they had received an OB infusion of VEH or SCOP (Investigation time = 29.9 ± 2.2 s and 33.4 ± 1.3 s, respectively). Together these experiments indicate that SCOP, administered either systemically or directly in the OBs, does not induce anosmia nor altered olfactory perception.

## Discussion

Using a combination of *in vivo* pharmacology and optogenetics in conjunction with olfactory fear conditioning, we investigated the role of ACh neurotransmission in olfactory fear learning. Previous work demonstrates that systemic administration of nicotinic AChR antagonists during conditioning does not alter fear learning, while systemic administration of mAChRs during acquisition suppresses olfactory fear learning (Pavesi et al., 2012). Therefore, we aimed to determine whether mAChRs specifically in the OB are required for acquisition of olfactory fear. The results demonstrate that acquisition, but not expression, of olfactory fear learning requires ACh neurotransmission through muscarinic receptors, specifically mAChR1, in the OB. Importantly, direct OB antagonism of mAChRs does not alter olfactory-driven behaviors, establishing that the lack of learning is not a consequence of reversible anosmia during olfactory fear conditioning. Finally, stimulating release of OB ACh during odor-shock pairing appears to increase the strength of fear learning. Together, these studies provide a new role for ACh in associative olfactory fear learning; however, the exact mechanism by which mAChR activation during odor-shock pairing allows for acquisition of fear learning remains unknown.

Our findings confirm previous reports that mAChRs are inextricably linked to olfactory learning. Both genetic (Chan et al., 2017) and pharmacological inhibition of mAChRs suppresses olfactory appetitive and aversive learning (Ravel et al., 1994; Kroon and Carobrez, 2009; Pavesi et al., 2012; Devore et al., 2014; Silva et al., 2015). This, combined with the present results, suggests that mAChRs play a similar role in acquisition of appetitive and aversive learning. We expand on these previous reports by demonstrating direct OB ACh signaling, specifically through mAChR1, is required during olfactory fear conditioning; however, the underlying mechanism remains unclear. One possibility is that the concentration of the mAChR1 antagonist (PIR) used (1 mM) is too high to exert a specific effect on m1 receptors and may be acting as an inverse agonist of m2 receptors (Daeffler et al., 1999); however, we find no effect of antagonizing mAChR2 receptors on learning at the same concentration. This suggests that even if PIR is modulating both mAChR1 and mAChR2 receptors at this concentration, the observed effect is specific to antagonism of mAChR1 receptors. ACh is known to modulate several OB cell types including MCs, granule cells (GCs), and periglomerular cells (Nickell and Shipley, 1988; Ravel et al., 1990; Castillo et al., 1999; Pressler et al., 2007; Chaudhury et al., 2009), but studies suggest that activation of mAChRs, especially mAChR1, increases excitability of GCs (Pressler et al., 2007; Smith and Araneda, 2010; Smith et al., 2015). Given olfactory learning is impeded when antagonism of mAChRs is confined to the GC layer but not affected when antagonism is confined to the glomerular layer (Ravel et al., 1994), mAChRs most likely modulate the MC/GC circuit, which increases synchronization of MC spike timing and oscillatory power (Li and Cleland, 2013). Models including pharmacological blockade of mAChRs in the OB result in altered OB network dynamics, which, in turn, decreases the activation of PCx, cortical plasticity, and learning (Devore et al., 2014). This may suggest the role of mAChRs during learning is to regulate olfactory input to PCx to enable olfactory learning. If this is the main function of mAChRs, muscarinic signaling in the OB is also likely required for appetitive learning, which future studies should investigate. While the exact downstream mechanisms are yet to be determined, activation of OB mAChRs in olfactory aversive learning likely regulates OB output, which ultimately leads to olfactory learning. Interestingly, our findings replicate previous reports that ACh modulation primarily affects acquisition but has little to no effect on expression of previous olfactory learning (Saar et al., 2001; Chapuis and Wilson, 2013; Linster and Cleland, 2016).

We also demonstrate that stimulating release of OB ACh during odor-shock pairing appears to increase the strength of fear learning. Optogenetic stimulation of OB ACh release cannot determine whether the facilitated learning is an effect of signaling through nicotinic AChRs, mAChRs, or both, and future studies are needed to determine the extent to which these different AChRs and their subtypes contribute to enhancement of olfactory fear learning. However, several previous reports establish that ACh release into the OB and subsequent activation of AChRs can modulate excitability of OB glomeruli and OB output cells (Chaudhury et al., 2009; Devore et al., 2012; Ma and Luo, 2012; Rothermel et al., 2014; Bendahmane et al., 2016). Enhanced synchrony and strengthened OB output could explain how optogenetically increasing OB ACh during odor-shock pairing results in strengthened olfactory learning. This idea is in line with previous reports that high ACh facilitates learning by enabling long term potentiation (Linster and Cleland, 2016). Another possibility is that OB ACh reduces the inhibitory drive of OB GCs, which could facilitate transmission of olfactory information from OB output neurons to higher processing centers (Elaagouby et al., 1991; Kay and Beshel, 2010; Kay, 2014; Osinski et al., 2018). Altering inhibitory drive of GCs could also lead to decreased inhibition of neighboring GCs (Castillo et al., 1999) thereby sharpening the receptive fields of OB output cells. It is also likely that activation of AChRs could inhibit glomerular layer inhibitory interneurons neurons (Crespo et al., 2000; Pignatelli and Belluzzi, 2008; Liu et al., 2015), leading to increased responses of excitatory cells. It is also possible that optogenetically stimulating cholinergic axons in the OB might produce back-propagation of action potentials to cholinergic soma, which could cause ACh release in multiple brain regions. Therefore, it is possible the facilitated learning following stimulated OB ACh release is actually due to non-specific ACh release. However, a recent study traced projections of basal forebrain cholinergic cells and found minimal cholinergic cells projecting to the main OB that also project to another region of the brain (Li et al., 2018), suggesting non-specific release is an unlikely explanation for the facilitated learning. Future electrophysiological experiments are needed to understand the exact role of ACh in facilitating olfactory aversive learning.

In summary, this study reveals new insights into the role of ACh in olfactory associative aversive learning. Previous reports using systemic antagonists indicated mAChRs, but not nicotinic AChRs, are required during odor-shock pairing for olfactory learning to take place. However, this did not address whether ACh signaling through mAChRs is required in the OB nor the extent to which olfactory aversive learning is mediated by specific mAChR subtypes. Using OB infusion of mAChR antagonists, we were able to determine that activation of mAChRs, specifically the m1 subtype, is required directly in the OB during odor-shock pairing for acquisition of olfactory fear learning. Additionally, we were able to confirm previous reports that blockade of mAChRs does not interfere with expression of previously learned olfactory fear. Furthermore, optogenetic stimulation of OB ACh during odor-shock pairing appears to increase the strength of olfactory learning. Together these studies demonstrate the importance of OB ACh for olfactory learning and related plasticity.

## Data Availability

The raw data supporting the conclusions of this manuscript will be made available by the authors, without undue reservation, to any qualified researcher.

## Ethics Statement

All experimental protocols were approved by the University of Tennessee Institutional Animal Care and Use Committee.

## Author Contributions

JR, MB, and MF designed the experiments. JR and MB performed all experiments, analyzed and interpreted the collected data. JR wrote the manuscript.

## Conflict of Interest Statement

The authors declare that the research was conducted in the absence of any commercial or financial relationships that could be construed as a potential conflict of interest.
